# Uncovering new MicroRNAs linked to acute pancreatitis: zeroing in on the protective effect

**DOI:** 10.1186/s41065-025-00607-0

**Published:** 2025-12-29

**Authors:** Changcheng Zhao, Shanshan Jia, Hang Yu, Lingtai Zhao, Yang Li, Jidong Zhang

**Affiliations:** Department of Gastroenterology, The First Hospital of Qiqihar City, Qiqihar, Heilongjiang China

**Keywords:** Acute pancreatitis, MicroRNA, Mendelian randomization, MiR-193a-5p; MiR-27b-3p

## Abstract

**Background:**

The microRNA expression profile in the bodily fluids of individuals with acute pancreatitis (AP) undergoes considerable alterations; nevertheless, the precise mechanism requires more elucidation.

**Methods:**

A dataset of 2083 human blood microRNAs (miRNAs) was obtained from the miRNA expression quantitative loci data. The genome-wide association study data for AP was obtained from the FinnGen Consortium. The causal association between miRNA and susceptibility to AP was evaluated using the Mendelian randomization (MR) method. Receiver operating characteristic (ROC) curve analyses were implemented to assess the miRNA’s clinical usefulness. The GSE188819 and GSE249349 datasets were analyzed to determine changes in cell subset amounts and differentially expressed genes during the advancement and regression of AP mice. We assessed the inhibitory impact of miR-27b-3p and miR-193a-5p overexpression on AR42J cell and RAW 264.7 cell inflammation using western blot.

**Results:**

Following a thorough process of genetic variation selection, MR analysis, and sensitivity analysis, we identified 66 miRNAs with suggestive causality to AP susceptibility. We conducted ROC curve analysis on 66 variables, identifying 6 miRNAs that have the potential to diagnose AP. Six and twelve cell subsets were identified from the GSE249349 and GSE188819 datasets, respectively. In the inflammatory advancement stage, the percentage of acinar cells in the AP group decreased relative to the control group samples. In the inflammatory regression phase, the percentage of monocytes in the AP (96 h) group decreased relative to the AP (12 h) group. In vitro, experiments have found that the overexpression of miR-27b-3p and miR-193a-5p in RAW 264.7 cells AR42J cells significantly inhibited the protein expression of p-P65.

**Conclusion:**

Our research identified novel miRNAs associated with the pathogenesis of AP. In vitro experiments have confirmed that miR-27b-3p and miR-193a-5p can inhibit the inflammatory response in RAW 264.7 cells and AR42J cells.

**Supplementary Information:**

The online version contains supplementary material available at 10.1186/s41065-025-00607-0.

## Introduction

Acute pancreatitis (AP) is a frequently encountered condition in the emergency department characterized by rapid progression [1]. Patients with mild AP (MAP) can undergo self-healing, whereas individuals with severe AP (SAP) frequently experience multiple organ dysfunction, posing a significant risk to their lives [1]. The worldwide incidence of AP stands at 34 cases per 100,000 individuals, showing a significant upward trend, while the age of onset is gradually decreasing [2]. Systemic inflammatory response syndrome (SIRS) and multiple organ failure, including acute respiratory distress syndrome and kidney failure, frequently coexist with SAP, posing significant challenges in clinical management [3, 4]. Despite the utilization of various pharmaceutical agents, including protease inhibitors, platelet-activating factor inhibitors, and immunomodulators, in managing patients with SAP, their efficacy remains limited, offering only partial delay in disease progression and inadequate improvement in patient prognosis [5–7]. The timely identification of the causative factors and underlying mechanisms can serve as a valuable point of reference for the clinical management of individuals affected by AP.

MicroRNAs (miRNAs) are a class of small, non-coding, single-stranded RNA molecules, typically consisting of 19 to 23 nucleotides. These molecules exhibit widespread expression across various tissues and cells. They undergo synthesis in the nucleus, where they are produced as precursor forms. Subsequently, these precursor forms are cleaved into mature forms within the cytoplasm. MiRNAs lack protein-coding capacity and primarily influence the translation process by selectively binding to the 3’ untranslated region of the target mRNA [8, 9]. A solitary miRNA can typically identify and attach to thousands of target mRNAs. This phenomenon explains the discrepancy between the small proportion of miRNAs in the human gene pool (2%) and their significant regulatory influence over approximately one-third of human genes. The miRNA expression profiles in tissues and body fluids of individuals with AP caused by various etiologies exhibited substantial alterations compared to those of healthy individuals. Observational study indicates a strong association between miRNA and the development of the disease, highlighting its potential as a diagnostic and prognostic marker [10]. Furthermore, several preclinical investigations have postulated that miRNA plays a role in developing AP by regulating various processes, such as the inflammatory cascade, programmed cell death, inflammatory cell infiltration, and intestinal barrier dysfunction. MiR-155 is a microRNA that is widely recognized for its pro-inflammatory properties. miR-155-5p has been reported to be upregulated during AP and may elicit intestinal barrier injury by targeting the suppressor of cytokine signaling 1-inflammasome signaling to initiate pyroptosis of epithelial cells [11]. An additional microRNA, miR-183-5p, was also substantially elevated in the plasma exosomes of AP patients, with its primary origin being the pancreatic acinar cells. MiR-183-5p may exacerbate the inflammatory response by downregulating forkhead box protein O1 expression, thereby promoting the M1-type polarization of macrophages [12]. It is important to note that microRNA also plays an antagonistic function in the development and occurrence of AP. In mice, the deletion of miR-29a increased pancreatic inflammation and fibrosis induced by caerulein [13]. Nevertheless, the limitations inherent in conventional observational studies, such as confounding variables and bias owing to reverse causality, have precluded the establishment of conclusive evidence regarding the causal association between miRNA and AP. The precise miRNA expression profile responsible for the development of AP remains unidentified.

Mendelian randomization (MR) studies employ genetic variation as an instrumental variable (IV), such as single nucleotide polymorphisms (SNPs), to evaluate the causal association between risk factors (exposure) and diseases (outcome). Katan first put out the idea in 1986 [14]. The study is grounded in Mendel’s second law of inheritance, which pertains to the impact of alleles on parental traits during gamete formation, resulting in their random allocation to offspring through meiosis. This procedure resembles randomized controlled trials (RCT), wherein individuals are randomly assigned to treatment and control groups. Therefore, MR has been hailed as a “natural RCT [15].” As more and more data from the Genome-Wide Association Study (GWAS) have been publicly available throughout the last few years, there has been a discernible rise in the use of the Mendelian randomization method [16]. This strategy has been successfully used in various contexts, including comprehending illness etiology, elucidating prospective diagnostic signs, and localizing treatment targets.

**Fig. 1:**
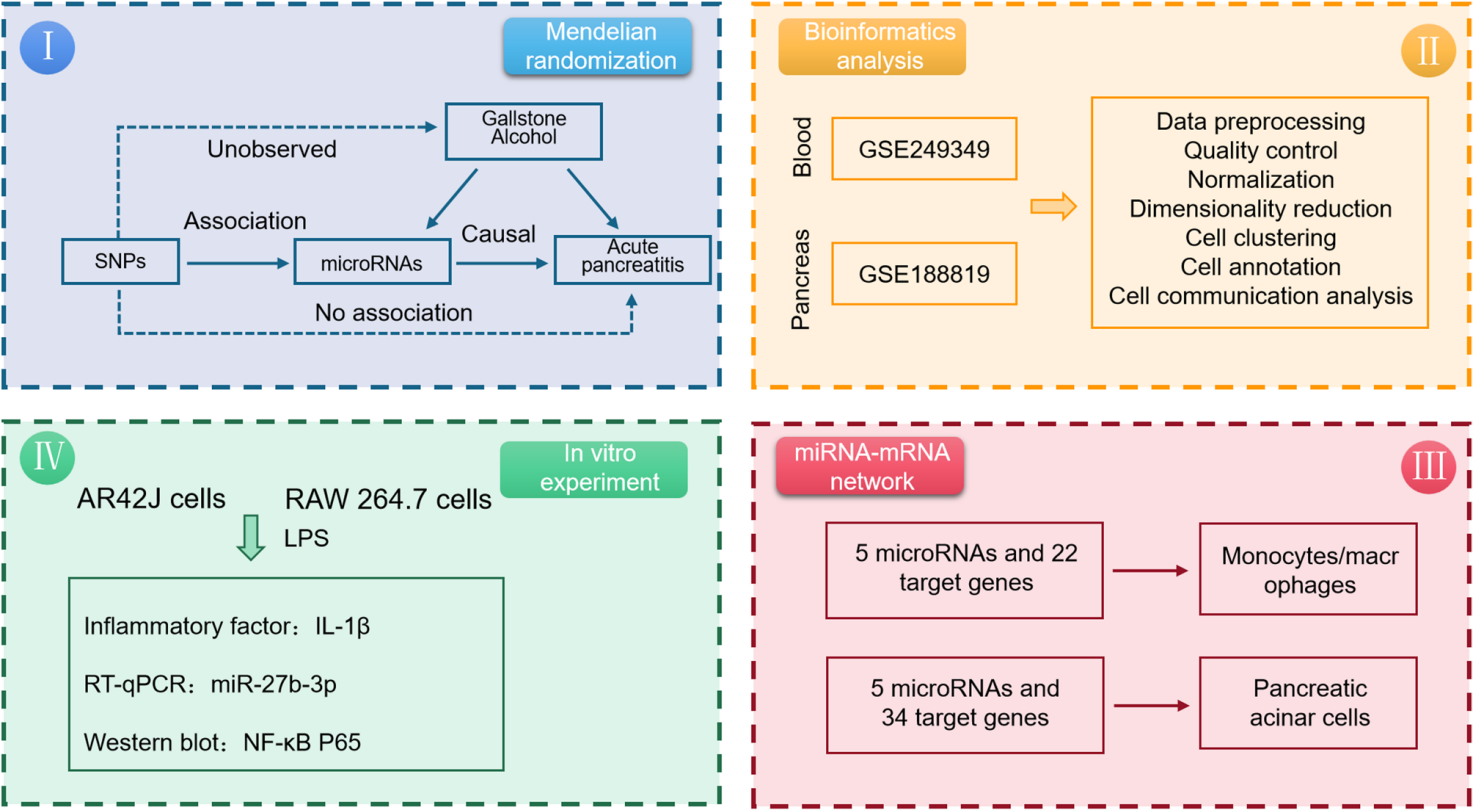
Flowchart of the study design. SNPs: single nucleotide polymorphisms

MR is considered epidemiology’s bedrock for establishing causal connections [17]. This study utilized the two-sample MR method to investigate the causal association between miRNA in peripheral blood and the risk of AP. Additionally, an in vitro AP model was developed to assess the inhibitory effects of specific miRNAs on cell damage and inflammation-these efforts aimed to enhance our comprehension of the underlying mechanisms involved in AP (Fig. 1).

## Methods and materials

### Research design and data source

Our MR study primarily comprises three sequential steps: The first involves carefully selecting suitable genetic variants. The second step entails investigating whether the genetic variants strongly associated with the exposure have causal effects on the outcomes. The third step investigated the biological association between miRNAs and AP. The exposure variable utilized in this study was miRNA, and the data were obtained from the largest miRNA expression quantitative trait loci (eQTLs) data [18]. The outcome data were acquired from the FinnGen Consortium. The AP dataset included 6,787 AP cases and 361,641 controls. The analysis was reported in compliance with the STROBE-MR recommendations (Supplementary Table S1) [19]. The data utilized in this study consists of publicly accessible and openly published information, thereby preventing the need for additional ethical clearance.

### Instrumental variable selection

The fundamental aspect of MR design lies in the selection and utilization of IVs. Reliable MR analysis relies on three fundamental assumptions: ①The correlation hypothesis (A strong correlation between the IVs and the exposure being studied), ②The independence hypothesis (IVs are not associated with any confounding factors), ③The hypothesis of exclusivity (IVs have the potential to influence outcomes solely through exposure). We selected the IVs based on a P-value threshold of less than 1E-05 (miRNA) or 5E-06 (AP). Linkage disequilibrium (LD) between SNPs can result in information overlap between the estimated effect sizes. Hence, we selected a LD parameter of r^2^ less than 0.001 and a region width of 10 MB to minimize the potential impact of gene pleiotropy on the causal estimation [20]. The utilization of SNPs with weak effects as IVs is associated with decreased precision and effectiveness of model estimation. As previously stated, a value of F-statistic more than 10 signifies a low probability of encountering a weak IV [21]. Concurrently, SNPs directly associated with AP were eliminated from consideration, with a statistical significance threshold of P-value less than 1 × 10^− 5^. The prevalent etiologies for AP are gallstone, alcohol consumption, hyperlipidemia, and autoimmune disease. We employed the PhenoScanner V2 website (http://www.phenoscanner.medschl.cam.ac.uk/) to ascertain SNPs linked to the aforementioned confounding factors. To ensure the robustness of the results, the SNPs associated with gallstones and alcohol consumption that correspond to a P-value less than 1 × 10^− 5^ are eliminated. Ultimately, miRNAs harboring three or more SNPs were selected for MR testing [22].

### MR analysis

The inverse variance weighting (IVW) method is widely used in MR analysis. The IVW method was employed as this study’s principal analytical approach to establish the causal association between miRNAs and AP. The MR-Egger, weighted median (WM), simple mode, and weighted mode methods are complementary to the IVW methods, providing additional support for the robustness of causal inference when both the direction and magnitude of the effects are consistent. The strength of the causal impact was expressed using odds ratio (OR) and a 95% confidence interval (CI). The Bonferroni correction approach is used to apply multiple testing adjustments in order to minimize the occurrence of false positives [23]. More precisely, significant causal effects are defined as those with a P-value of less than 2.4 × 10^− 5^ (0.05/2083, with 2083 tests conducted), those with a P-value between 0.05 and 2.4 × 10^− 5^ as suggestive statistical significance, and those with a P-value greater than 0.05 as insignificant. Cochran’s Q test assessed heterogeneity among IVs [24]. The MR-Egger intercept is employed to identify pleiotropy [25]. Using the leave-one-out (LOO) method, it is possible to ascertain whether a solitary SNP influences the causal relationship. The statistical analysis used R version 4.1.2 with the “TwoSampleMR” package.

### Analysis of single-cell RNA sequencing data

The single-cell RNA sequencing data of peripheral blood and pancreatic tissues of AP mice were obtained from the GEO database. The downstream analysis is conducted based on the Seurat package. Low-quality cells were filtered through percent.mt, nCount_RNA, and nFeature_RNA. The NormalizeData and ScaleData functions are adopted to normalize and standardize the data, and default parameters are used for both. Use the RunUMAP function for dimensionality reduction analysis. Use the harmony package to correct the batch effect in different samples. Cell clustering was performed using the FindClusters function (resolution = 1), and the FindAllMarkers function was used to screen for differentially expressed genes (DEGs) for each cluster. According to DEGs, cell subsets were annotated using the SingleR package and the CelMarker database. Draw a stacked bar chart of cell subset ratios in different samples. Further use CellChat to analyze the intercellular communication network.

### MiRNA-mRNA network construction

The target genes of miRNA were obtained from the TargetScanHuman 8.0 (https://www.targetscan.org/vert_80/), miRDB (https://mirdb.org/) and miRTarBase (https://mirtarbase.cuhk.edu.cn/) databases, respectively. Only the target genes common to the three databases were retained to enhance the dependability of the findings. Retrieve the GSE194331 dataset (https://www.ncbi.nlm.nih.gov/geo/query/acc.cgi?acc=GSE194331) about AP from the GEO database. The dataset comprised 57 individuals diagnosed with AP and 32 healthy controls. Specific criteria were employed to identify differentially expressed genes (DEGs) in the peripheral blood of individuals with AP compared to healthy controls. Specifically, the absolute value of the Log_2_ fold change had to be greater than 0.58, and the P-value had to be less than 0.05. We preserved the genes common to DEGs and miRNA target genes. The miRNA-mRNA network was mapped, followed by topological analysis. Receiver operating characteristic (ROC) curve analyses were implemented to assess the miRNA’s clinical usefulness.

### Cell culture and treatment

Mouse mononuclear macrophages (RAW 264. 7 cells) were grown at a constant temperature of 37 ℃ and 5% CO_2_ using RPMI 1640 supplemented with 10% FBS and 1% penicillin-streptomycin. Upon achieving a cell density of 70% − 80%, the cells were subjected to digestion with 0.25% trypsin and then subcultured for further experimentation. Rat pancreatic acinar cells (AR42J) were grown in DMEM media supplemented with 10% fetal bovine serum, 1% penicillin, and 1% streptomycin. The cells were incubated at 37 ℃ and a CO_2_ concentration of 5%. The culture media was replaced every 2–3 d, and cell passage was performed when the number of cells adhered to the wall exceeded 80%. The AR42J cells were used for further investigations in the logarithmic growth phase. The cells were categorized into four groups: (1) control (CON) group, (2) Lipolyaccharide (LPS) group (treated with 2 µg/ml LPS for 24 h), (3) LPS + miR-193a-5p mimics group, (4) LPS + miR-27b-3p mimics group. The concentrations of miR-193a-5p and miR-27b-3p mimics (GenePharma, China) were both diluted to 10 nmol/L.

### qRT-PCR

Total RNA was isolated from RAW264.7 cell precipitates using the TRIzol technique. Reverse transcription is performed in accordance with the kit’s instructions. Conduct the test on a single sample three times. A relative quantitative analysis was performed using the 2^−ΔΔCt^ technique, with U6 serving as the internal reference [26].

### Western Blot (WB)

Cells from each group were harvested, and RIPA lysis buffer with protease inhibitor was added, followed by incubation on ice for 30 min to achieve complete lysis. Following centrifugation, the supernatant was collected, and the protein content was assessed. Introduce the sample buffer and denature at 100℃ for 10 min. The protein was isolated using 10% SDS-PAGE, followed by transfer and block, after which primary antibodies p-P65 and P65 were added and incubated at 4℃ overnight. Subsequent to the removal of the first antibody the next day, the second antibody was introduced at ambient temperature, incubated for 2 h, and the membrane was then washed again. The target protein expression was assessed using the chemiluminescence technique, and the grey value was analyzed using ImageJ software.

### Detection of Interleukin-1 β (IL-1β)

The culture supernatants of AR42J cells in each group were collected into centrifuge tubes, and an appropriate amount of protease inhibitor was added. Centrifugation was carried out at 2000 g for 20 min, and the supernatants were taken to detect the content of IL-1β. The detection steps were carried out according to the instructions of the kit.

### Statistical analysis

The experimental results were presented as the mean ± standard deviation. Statistical analysis was performed using R studio (v 4.3.1), and a one-way analysis of variance was employed to compare several groups. A *P*-value < 0.05 was deemed to be statistically significant.

## Results

### Causal effects of MiRNAs on AP

**Fig. 2:**
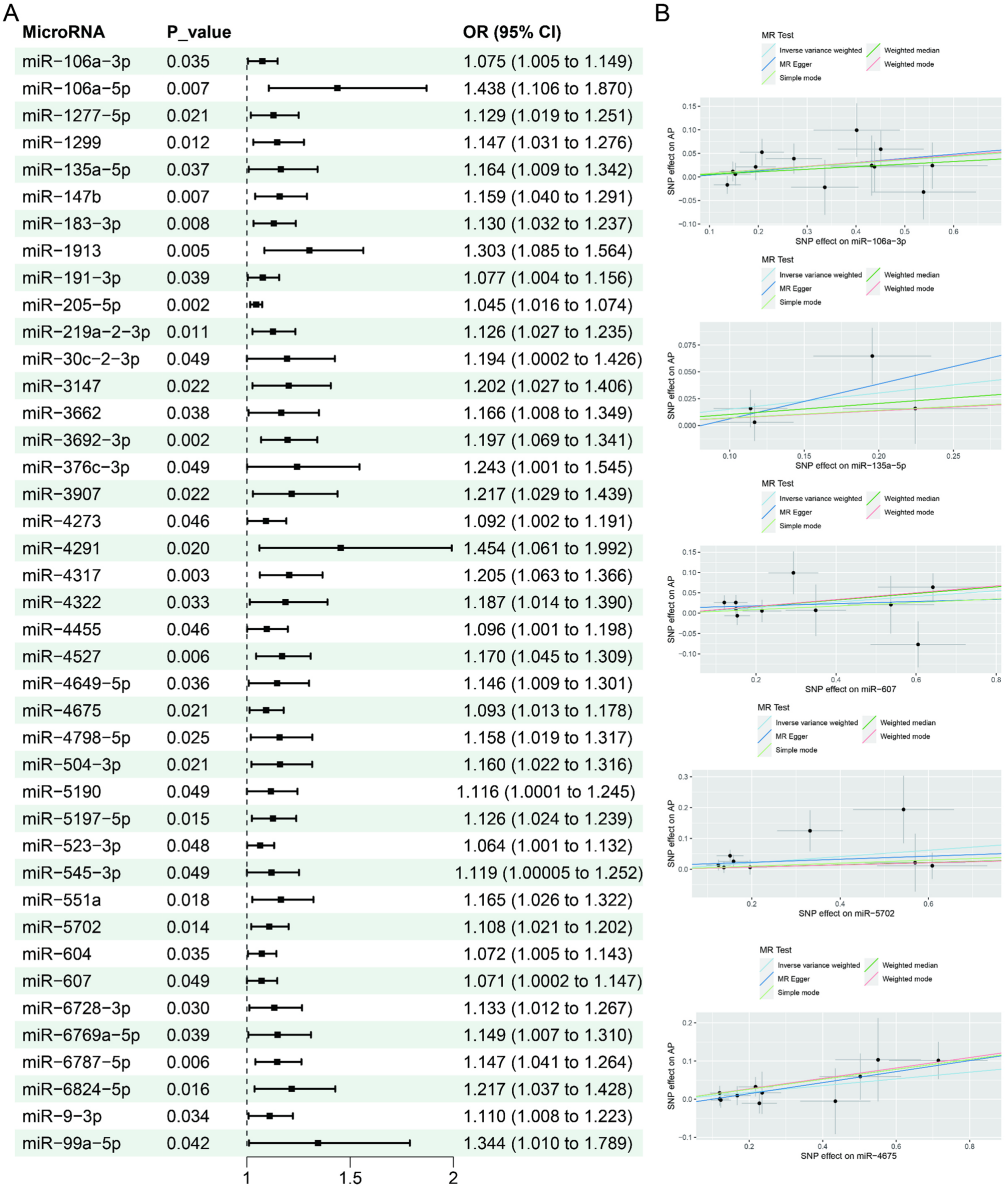
Forest plots and scatter of causal effects of miRNAs (odds ratio > 1) on acute pancreatitis. SNPs: Single Nucleotide Polymorphisms, OR: Odds Ratio, CI: Confidence Interval

**Fig. 3:**
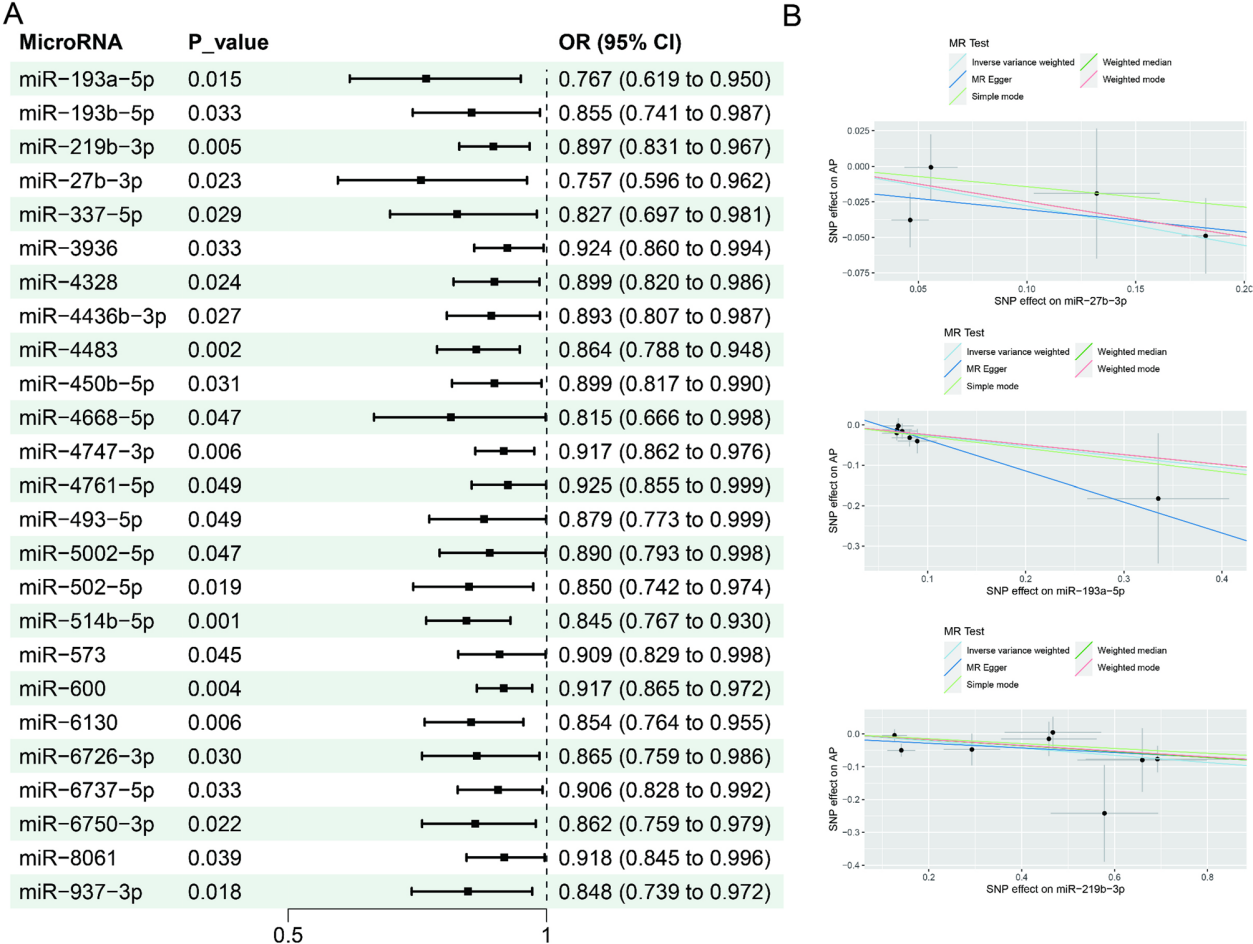
Forest plots and scatter of causal effects of miRNAs (odds ratio < 1) on acute pancreatitis. SNPs: Single Nucleotide Polymorphisms, OR: Odds Ratio, CI: Confidence Interval

In brief, a suggestive causal association was identified between 66 miRNAs and AP risk using random-effects IVW methods (*P*-values were all between 2.4 × 10^− 5^ and 0.05). In particular, a positive association was observed between 41 miRNAs and the risk of AP (Fig. 2). 25 miRNAs exhibited a negative correlation with the risk of AP (Fig. 3). It is worth mentioning that in the reverse MR analysis of AP with the above 66 miRNAs, four miRNAs (miR-6769a-5p, miR-106a-5p, miR-193a-5p and miR-4455) showed significance. For specific correlation data, please refer to Supplementary Table S2.

Cochran’s Q test suggested the absence of heterogeneity. Furthermore, the MR-Egger regression analysis indicates that pleiotropy is absent, as shown in Supplementary Table S3. The findings above demonstrate the robustness of the causal estimation obtained in this study.

### Assessment of the clinical usefulness

We used previously published miRNA expression profiles to get expression data for 66 miRNAs from blood exosomes from AP patients and healthy volunteers [27]. We conducted ROC curve analysis on 66 variables, identifying 6 miRNAs (AUC >0.65) that have the potential to diagnose AP, as shown in Supplementary Figure S1.

### Analysis of single-cell RNA sequencing data from peripheral blood in AP mice

**Fig. 4:**
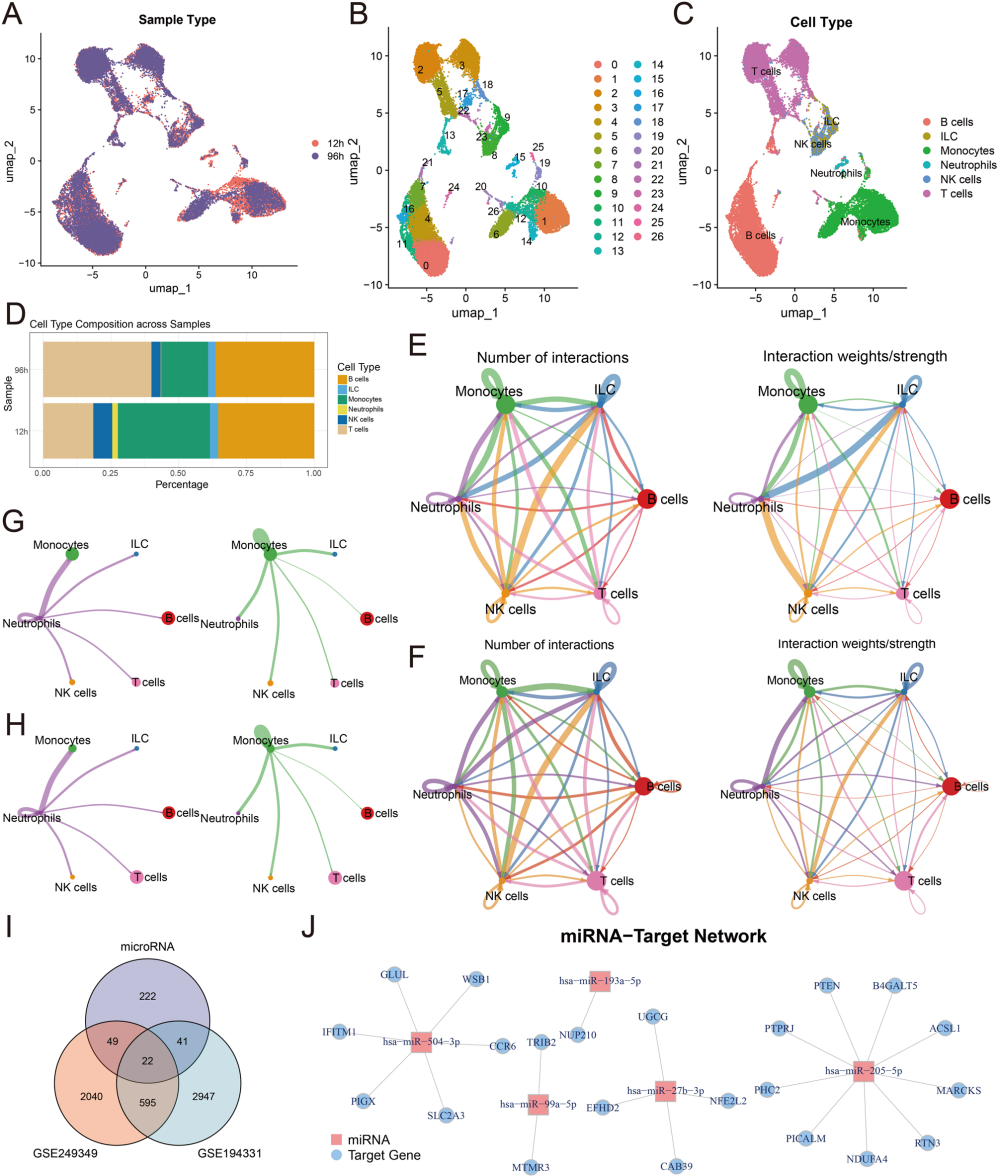
Analysis of the GSE249349 dataset. **A** UMAP plot showing the sample distribution at 96 h and 12 h. **B** The UMAP plot showing the major classifications. **C** The UMAP plot showing the main cell types. **D** The relative proportions of cell populations in the samples at 96 h and 12 h. **E** Crosstalk analysis between 12 h sample cells. **F** Crosstalk analysis between 96 h sample cells. **G** Crosstalk analysis of monocytes in the 12 h sample with other cell types. **H** Crosstalk analysis of 96 h sample monocytes with other cell types. **I** Intersection analysis of target genes. **J** Construction of miRNA-mRNA networks in monocytes

Single-cell RNA sequencing profiles of peripheral blood from AP mice were produced using GSE249349 data. Following interventions including quality control, normalization, dimensionality reduction, and batch effect elimination, 25,485 cells were preserved (Fig. 4A). Cell annotation was conducted on 27 subgroups, resulting in the identification of 6 cell groups: B cells, ILC, Monocytes, Neutrophils, NK cells, and T cells (Fig. 4B&C). Construct the bar chart illustrating the percentage of each cell type across two samples (Fig. 4D). In comparison to the 12-h sample, the percentage of monocytes in the 96-h sample dropped considerably. CellChat elucidated the relationships among various cell subsets. The findings indicated that during the inflammation regression of AP, the interaction events between monocytes and other cell subsets were markedly reduced (Fig. 4E-H). Due to the pivotal function of monocytes/macrophages in AP, monocytes were chosen for more examination. A total of 2,706 DEGs were discovered. Additionally, 3,605 DEGs were discovered in the GSE194331 database. Following the intersection analysis of the aforementioned DEGs with 334 miRNA target genes, a total of 22 common genes were identified (Fig. 4I). We further developed the miRNA-target gene network shown in Fig. 4J.

### miR-193a-5p and miR-27b-3p inhibit LPS-induced inflammation in RAW264.7 cells

**Fig. 5:**
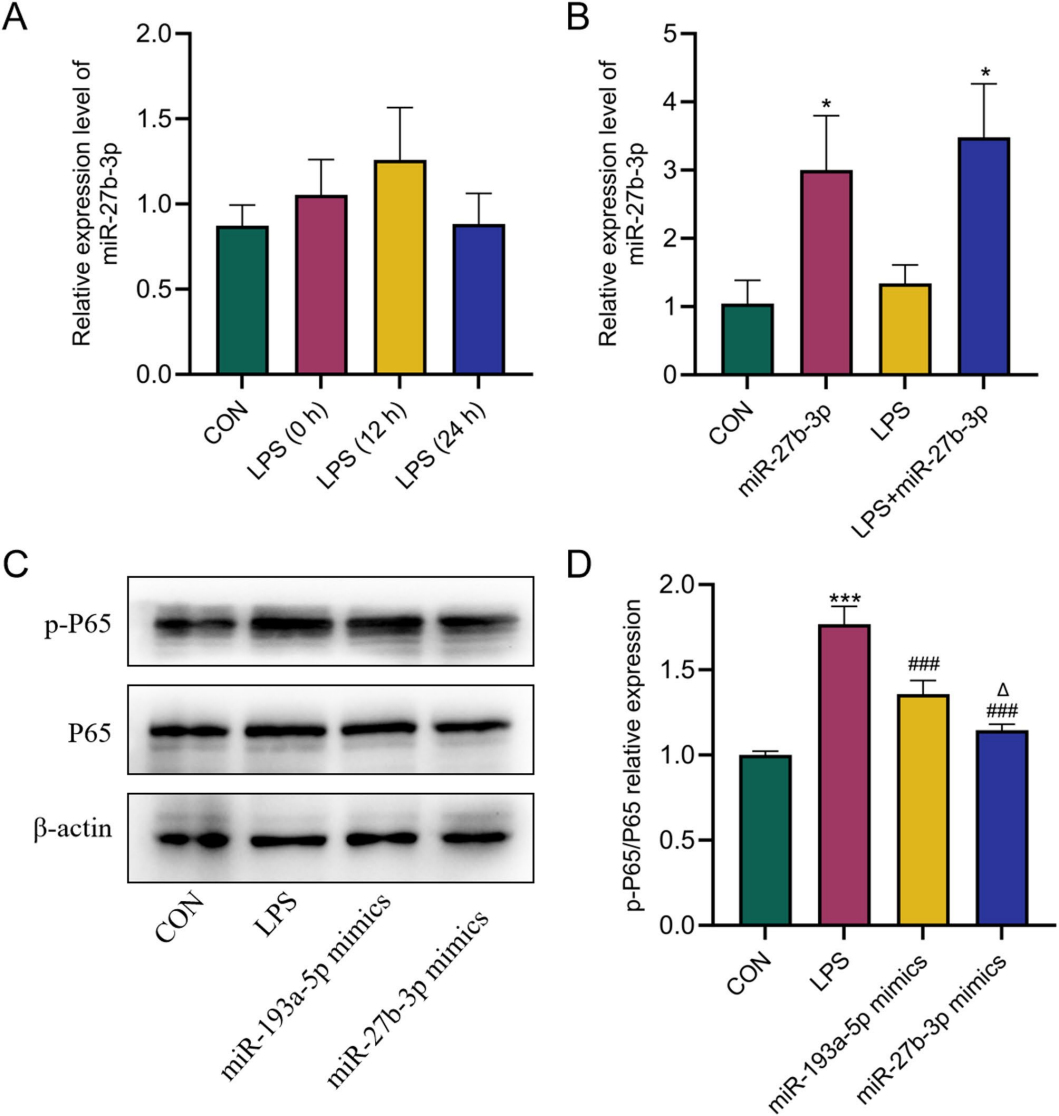
Effect of miR-27b-3p on LPS-induced inflammation of RAW264.7 cells. **A**, **B**. The relative expression level of miR-27b-3p. **C**, **D** Overexpression of miR-27b-3p significantly reduced the expression of p-P65 (*n* = 3). Data are expressed as mean ± SD. ^*^ means *P*-value < 0.05 compared with CON group. ^*^ means *P*-value < 0.05 compared with LPS group. ^***^ means *P*-value < 0.001 compared with CON group. ^##^ means *P*-value < 0.01 compared with NC group. ^###^ means *P*-value < 0.001 compared with LPS group. ^△^ means *P*-value < 0.05 compared with miR-193a-5p mimics

We selected miR-27b-3p for further experimental verification because of its clinical value (AUC = 0.778) and protective effect (OR = 0.757). We induced inflammation in RAW264.7 cells through LPS. Initially, we assessed the expression level of miR-27b-3p in RAW264.7 cells at various temporal intervals. Consistent with other research [26], we observed that LPS stimulation did not elicit the over-expression of miR-27b-3p in RAW264.7 cells (Fig. 5A). Nonetheless, miR-27b-3p mimics may markedly enhance the expression level of miR-27b-3p in RAW264.7 cells (Fig. 5B). Previous studies suggested that miR-193a-5p is a miRNA that inhibits AP, so we chose it as the control [28]. NF-κB is a prominent transcription factor that induces pro-inflammatory responses in macrophages. WB results showed that overexpression of miR-27b-3p and miR-193a-5p significantly decreased the expression of NF-κB p-P65 protein compared with LPS group. The impact of miR-27b-3p on inflammation is much superior than that of miR-193a-5p (Fig. 5C&D).

### Analysis of single-cell RNA sequencing data from pancreatic tissue in AP mice

**Fig. 6:**
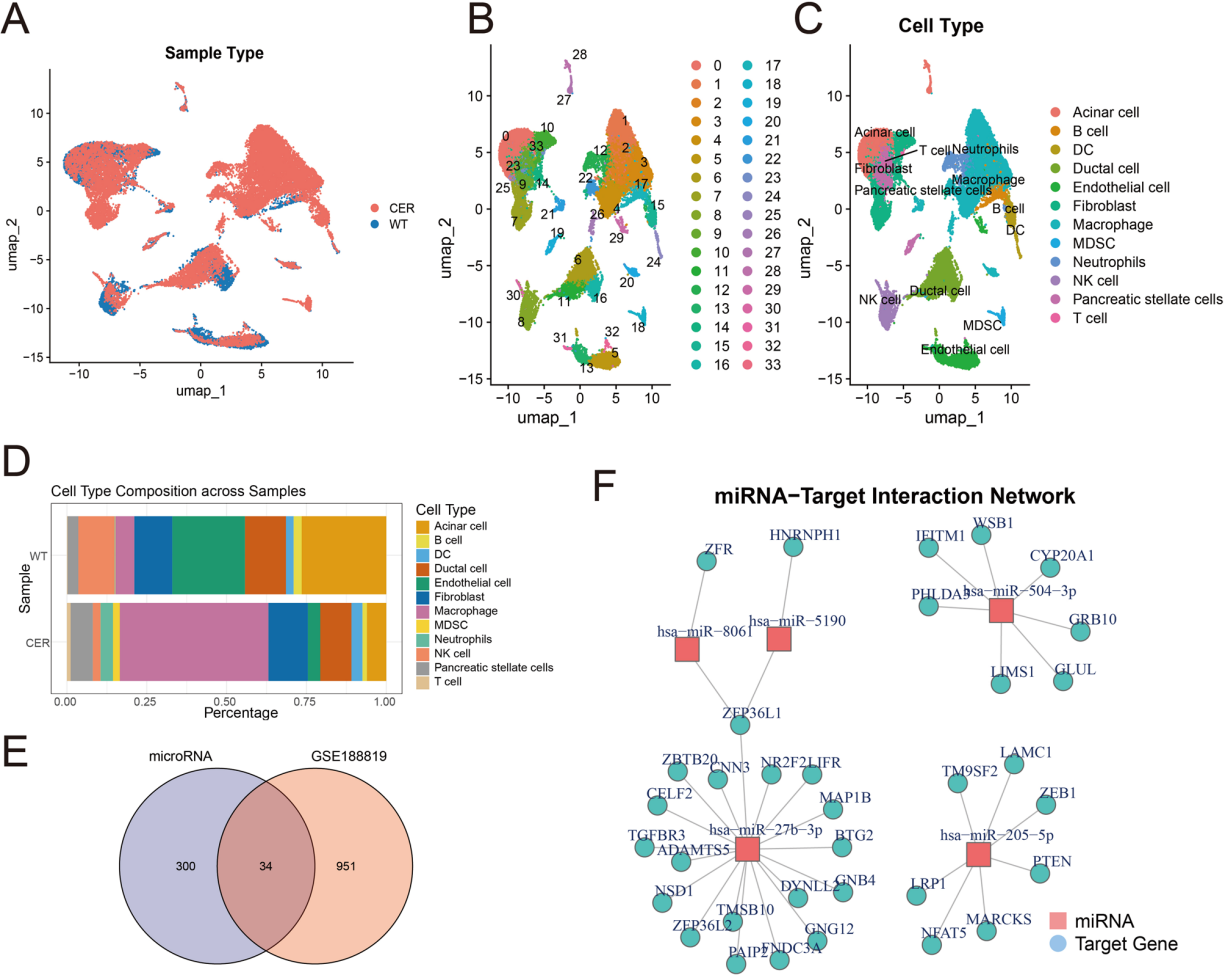
Analysis of the GSE188819 dataset. **A** UMAP plot showing the sample distribution of CER and WT samples. **B** The UMAP plot showing the major classifications. **C** The UMAP plot showing the main cell types. **D** The relative proportions of cell populations in the CER and WT samples. **E** Intersection analysis of target genes. **F** Construction of miRNA-mRNA networks in acinar cells

Maps of single-cell RNA sequencing of pancreatic tissue in AP mice were produced using GSE188819 database. Following interventions including quality control, normalization, dimensionality reduction, and batch effect elimination, 29,362 cells were preserved (Fig. 6A). Cell annotation was conducted on 34 subgroups, resulting in the identification of 12 principal cell groups: Acinar cell, B cell, dendritic cell (DC), ductal cell, endothelial cell, fibroblast, macrophage, myeloid-derived suppressor cell, neutrophils, natural killer (NK) cell, pancreatic stellate cells, and T cell (Fig. 6B&C). Construct the bar chart illustrating the percentage of each cell type across various samples (Fig. 6D). In comparison to the WT sample, the percentage of macrophages in the CER sample exhibited a considerable rise, but the fraction of acinar cells shown a significant drop. Given the pivotal function of acinar cells in AP, these cells were chosen for further examination. A total of 985 DEGs were discovered. Following the intersection analysis of 334 miRNA target genes, a total of 34 common genes were identified (Fig. 6E). We also developed the miRNA-target gene network shown in Fig. 6F.

### miR-193a-5p and miR-27b-3p inhibit LPS-induced inflammation in AR42J cells

**Fig. 7:**
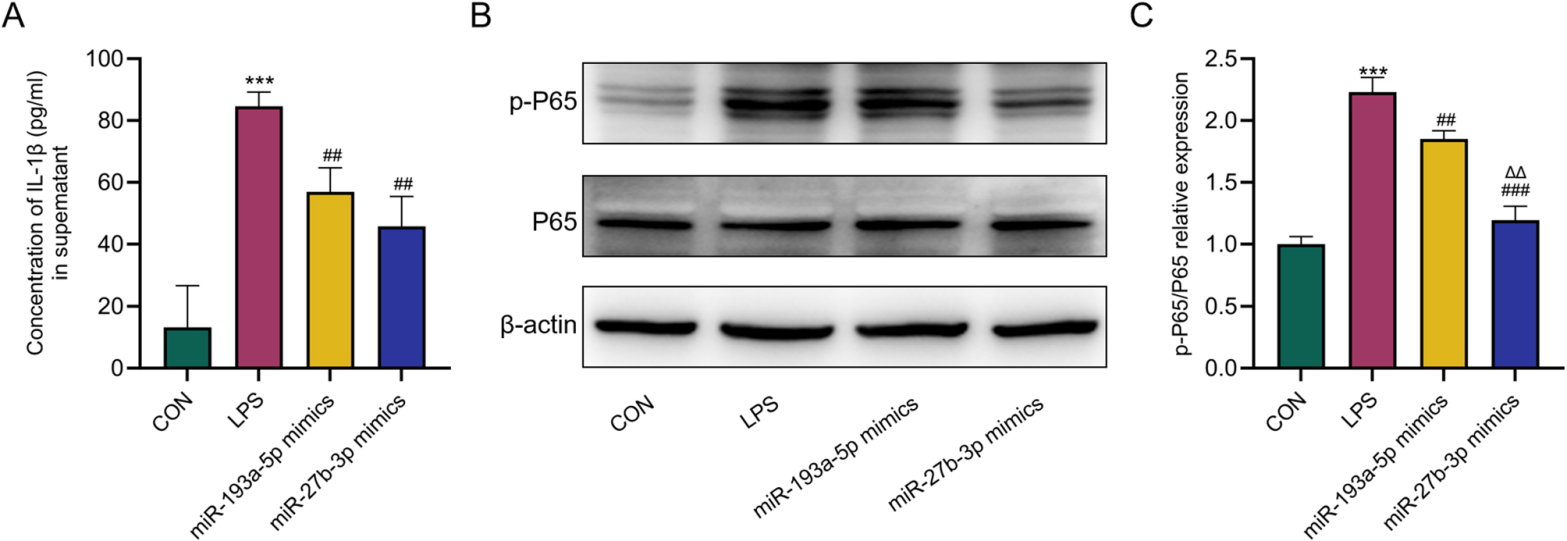
Effect of miR-27b-3p on LPS-induced inflammation of AR42J cells. **A** Overexpression of miR-27b-3p significantly reduced the expression levels of IL-1β (*n* = 3); **B**, **C** Overexpression of miR-27b-3p significantly reduced the expression of p-P65 (*n* = 3). Data are expressed as mean ± SD. ^***^ means *P*-value < 0.001 compared with CON group, and ^##^ means *P*-value < 0.01 compared with LPS group. ^###^ means *P*-value < 0.001 compared with LPS group. ^△△^ means *P*-value < 0.01 compared with miR-193a-5p mimics

We induced inflammation in AR42J cells through LPS. The ELISA data indicate that LPS caused an inflammatory response in AR42J cells, primarily characterized by elevated levels of IL-1β (Fig. 7A). Nevertheless, the over-expression of miR-27b-3p and miR-193a-5p markedly reduced the concentration of IL-1β. WB results showed that overexpression of miR-27b-3p and miR-193a-5p significantly decreased the expression of p-P65 protein compared with LPS group. The impact of miR-27b-3p on inflammation is much superior than that of miR-193a-5p (Fig. 7B&C).

## Discussion

AP is a frequently encountered abdominal condition. Individuals diagnosed with mild AP exhibit relatively mild symptoms and have the potential to undergo spontaneous resolution [29]. While the prevalence of SAP patients is relatively low, it is noteworthy that both the mortality and complication rates associated with this condition are significantly elevated [30]. Moreover, AP exhibits a propensity for relapse and has the potential to advance into chronic pancreatitis, thereby imposing a substantial financial and healthcare burden on both medical insurance systems and affected individuals. MiRNA is a category of small non-coding RNAs that exhibit high conservation and are found in various tissues and organs, displaying notable specificity. MiRNAs are detectable in various bodily fluids and can serve as diagnostic markers and therapeutic targets [8, 31]. While specific literature has suggested a potential link between aberrant miRNA expression patterns and the pathogenesis of AP, the current body of evidence lacks sufficient quality to establish a definitive causal relationship between miRNA and the development of AP [10]. Through the utilization of GWAS data and the MR method, 66 miRNAs has been identified as having a suggestive causal association with the risk of AP. Furthermore, reverse MR analysis suggested that AP may have possible causal relationships with 4 of the 66 aforementioned miRNAs. We hypothesise that there may be a self-reinforcing positive feedback loop involving these four miRNAs and AP. On the other hand, this bidirectional correlation may be attributable to shared genetic confounding variables rather than a genuine causal relationship. Consequently, other evidence must be included to validate the aforementioned link.

Therefore, we utilized external data to evaluate the hypothesis. Exosomes frequently serve as carriers for miRNA transport, a widely recognized fact. Qu et al. previously described blood-derived exosomal miRNA expression profiles in three AP patients and three healthy volunteers [27]. Consequently, we extracted the expression data of 66 miRNAs from the blood-derived exosomes of the two groups of individuals. We identified six miRNAs as possible diagnostic markers: hsa-miR-27b-3p, hsa-miR-99a-5p, hsa-miR-193a-5p, hsa-miR-205-5p, hsa-miR-504-3p, and hsa-miR-8061. This result is derived on a research with an inadequately small sample size. Future multi-center studies with larger sample sizes are necessary to assess the diagnostic efficacy of these miRNAs.

We included the GSE249349 and GSE188819 datasets to analyze the single-cell RNA expression profiles in the peripheral blood and pancreatic tissues of AP mice. We identified six cell subsets in the peripheral blood samples of AP mice. During the period of inflammation resolution, the number of monocytes decreased significantly. We identified 334 target genes from 6 miRNAs. After intersection processing with the DEGs of the GSE194331 and GSE249349 datasets, we constructed a network including 5 miRNAs and 22 target genes. Among them, miR-27b-3p is the core miRNA. Furthermore, we identified 12 cell populations in the pancreatic tissues of AP mice. During the progressive stage of inflammation, we found that the number of acinar cells decreased significantly. Furthermore, we identified 985 DEGs from acinar cells. After intersection processing with 334 miRNA target genes, a network including 5 miRNAs and 34 target genes was constructed. Similarly, miR-27b-3p is the core miRNA.

Without other studies showing the relationship between miR-27b-3p and AP, our findings reveal that hsa-miR-27b-3p may demonstrate remarkable protective properties. Although previous study showed that the impact of miR-27b-3p on inflammation exhibits conflicting outcomes. One potential mechanism by which hsa-miR-27b-3p may mitigate TNF-α-induced endothelial dysfunction and mitochondrial dysfunction is through the activation of the forkhead box O pathway [32]. In contrast, it has been observed that miR-27b-3p can effectively enhance endothelial inflammation in the context of atherosclerosis. This phenomenon is likely attributed to the suppression of the PPARα signaling pathway [33]. Furthermore, it has been observed that miR-27b-3p exhibits distinct regulatory effects on various macrophage subtypes [34, 35]. During the pathogenesis of AP, endothelial cells are transformed into a pro-inflammatory phenotype, which subsequently facilitates the accumulation of mononuclear macrophages and neutrophils within the pancreatic tissue [36]. Several previous studies have reported the inhibitory effect of miR-193a-5p on AP-induced inflammation, which may be related to the regulation of pyroptosis [28, 37, 38].

Given the potential protective impact of miR-27b-3p and miR-193a-5p, we investigate whether increasing miR-27b-3p and miR-193a-5p expression might reduce the occurrence and progression of AP. In vitro experiments shown that elevated levels of miR-27b-3p and miR-193a-5p could suppress LPS-induced inflammation in RAW264.7 and AR42J cells. Based on MR studies and in vitro experiments, we have found evidence suggesting that miR-27b-3p and miR-193a-5p may be a protective factor for AP. Notably, miR-27b-3p has a much greater impact on inflammation than miR-193a-5p.

We employed a variety of methodologies to validate the reliability and stability of the results obtained from the MR analysis. Despite the acquisition of solid evidence in this study, certain limitations persist. This study incorporated 2083 miRNAs for MR analysis. We recommended that future studies include larger sample sizes to enhance the precision of identifying miRNAs that exhibit causal associations with AP. Furthermore, the research was conducted using European populations did not fully consider genetic variation between different ethnic groups. The findings derived from this study solely offer a theoretical framework for understanding the causal association between miRNA and AP.

## Conclusion

The present study employed MR methods to investigate the causal association between peripheral blood miRNA and AP. We identified 66 miRNAs with suggestive causality to AP susceptibility and 6 miRNAs that have the potential to diagnose AP. We also discovered that the overexpression of miR-27b-3p and miR-193a-5p in LPS-treated AR42J and RAW264.7 cells significantly inhibited cellular inflammatory responses. This discovery enhances our comprehension of the pathogenesis of AP.

## Supplementary Information

Supplementary Material 1:Supplementary Figure S1.ROC analysis. Individual ROC curves for hsa-miR-27-3p (A), hsa-miR-99a-5p (B), hsa-miR-193a-5p (C), hsa-miR-205-5p (D), hsa-miR-504-3p (E), and hsa-miR-8061 (F).

Supplementary Material 2: Supplementary Table S1. STROBE-MR checklist.

Supplementary Material 3: Supplementary Table S2. Mendelian randomization analysis.

Supplementary Material 4: Supplementary Table S3. Sensitivity analysis for the causal association between miRNAs and AP.

Supplementary Material 5.

## Data Availability

No datasets were generated or analysed during the current study.

## Abbreviations

AP: Acute pancreatitis
SIRS: Systemic inflammatory response syndrome
MiRNAs: MicroRNAs
MR: Mendelian randomization
IV: Instrumental variable
SNP: Single nucleotide polymorphisms
RCT: Randomized controlled trials
GWAS: Genome-Wide Association Study
LD: Linkage disequilibrium
IVW: Inverse variance weighting
WM: Weighted median
OR: Odds ratio
CI: Confidence interval
LOO: Leave-one-out
ROC: Receiver operating characteristic
GO: Gene Ontology
KEGG: Kyoto Encyclopedia of Genes and Genomes
CON: Control
LPS: Lipolyaccharide
ELISA: Enzyme-linked immunosorbent assay
IL: Lnterleukin
WB: Western blot
BP: Biological process
CC: Cellular component
MF: Molecular function
