# Supplementary figures and images for: Uncovering new MicroRNAs linked to acute pancreatitis: zeroing in on the protective effect
Source: Hereditas. 2025 Dec 29;162:238. doi: 10.1186/s41065-025-00607-0 (PMC12751974; doi:10.1186/s41065-025-00607-0)

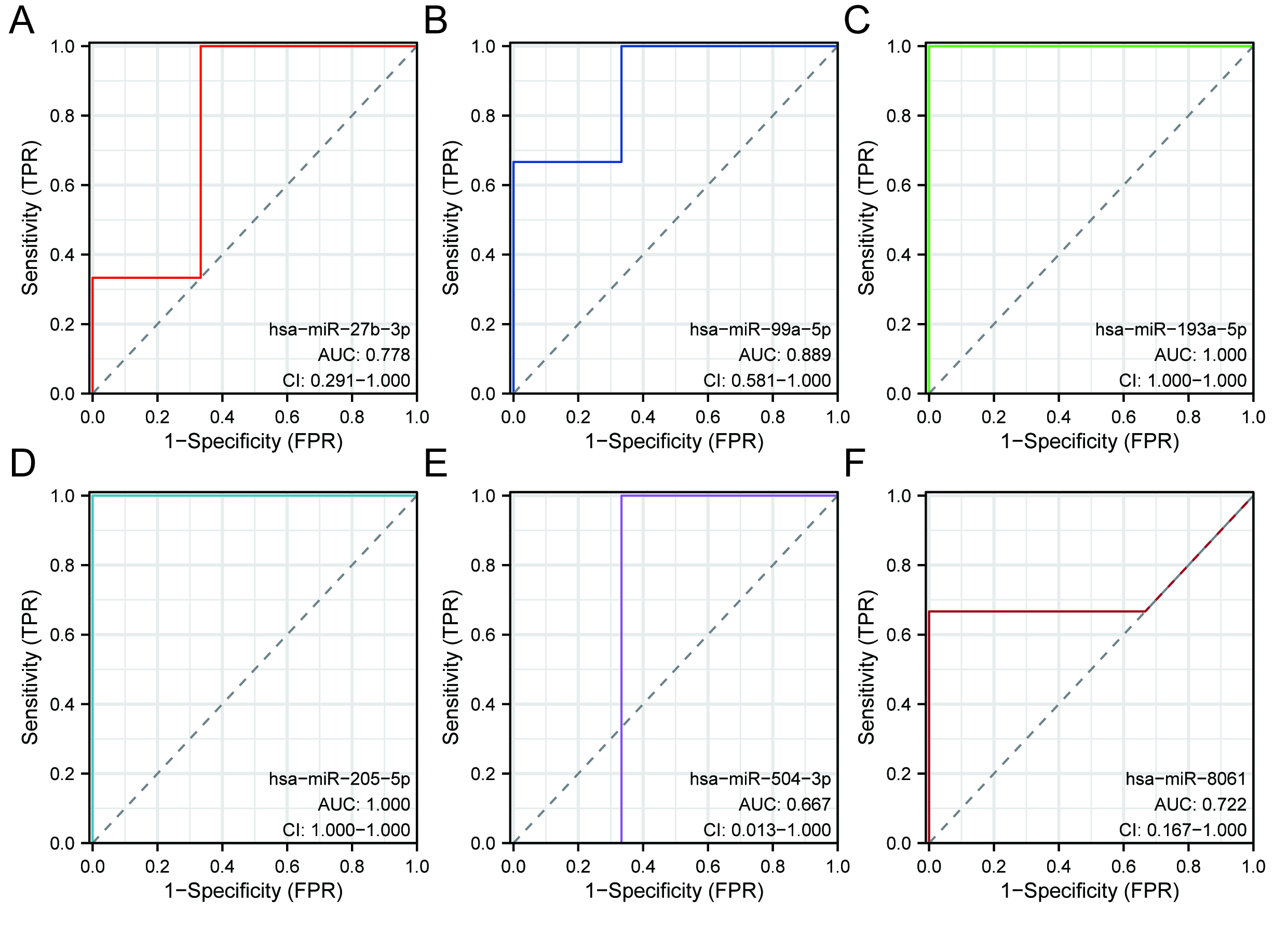

Supplement: Supplementary file 1 — Supplementary Material 1:Supplementary Figure S1.ROC analysis. Individual ROC curves for hsa-miR-27-3p (A), hsa-miR-99a-5p (B), hsa-miR-193a-5p (C), hsa-miR-205-5p (D), hsa-miR-504-3p (E), and hsa-miR-8061 (F). [file 41065_2025_607_MOESM1_ESM.jpg]

Figure 6A

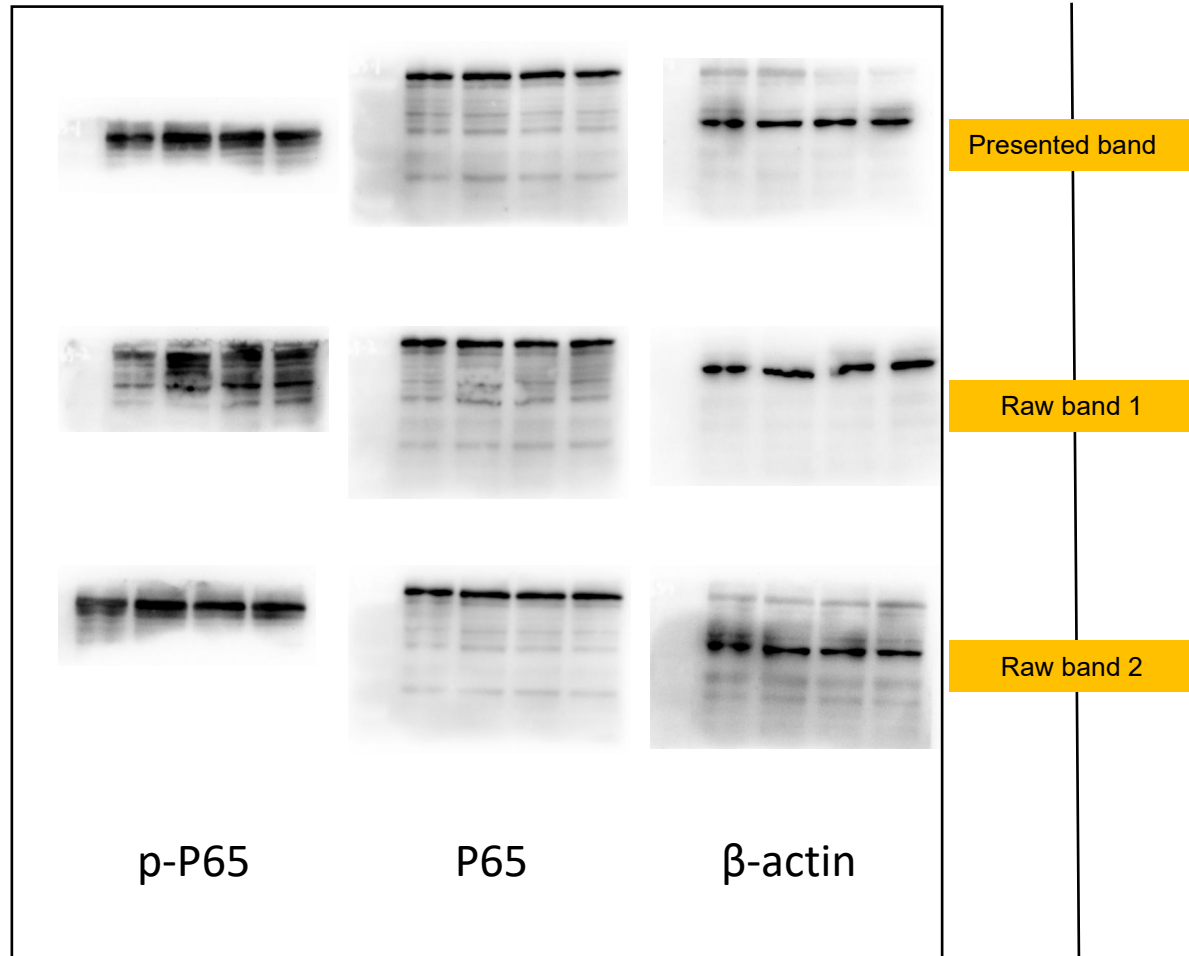

Figure 8B

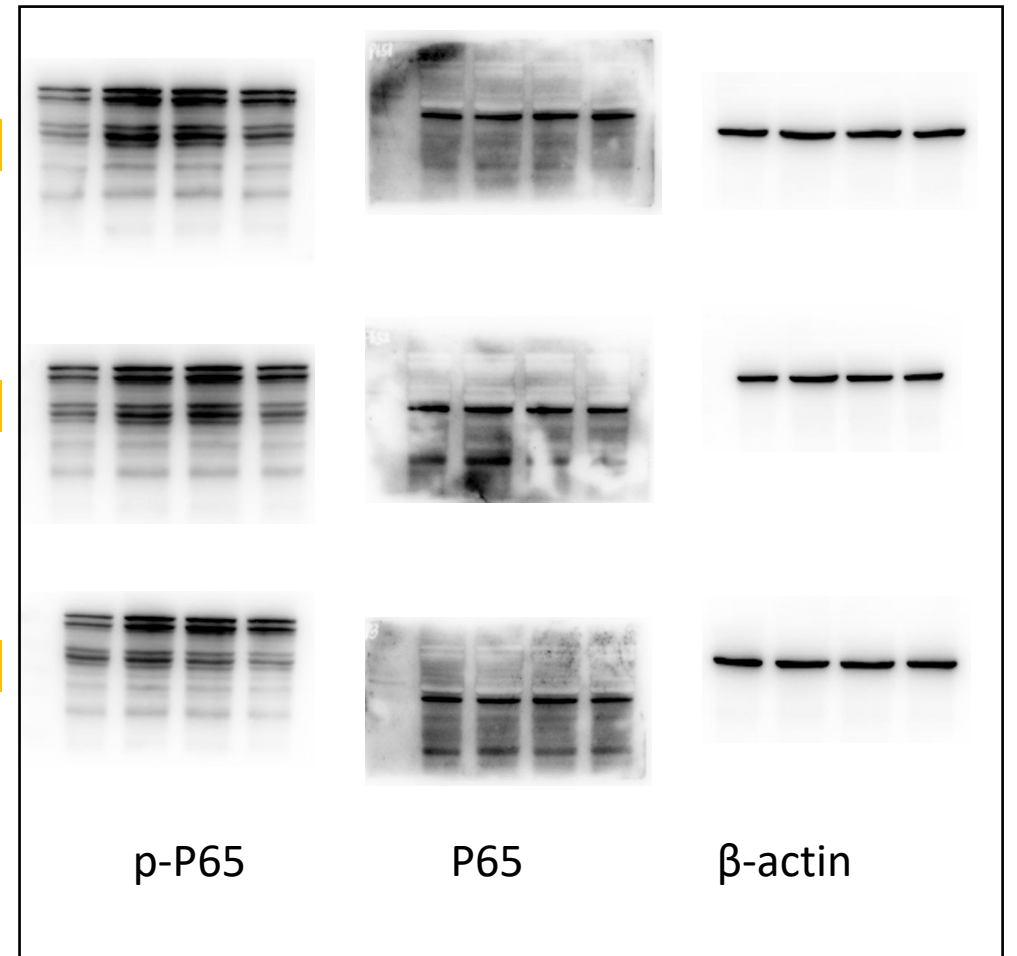

Supplement: Supplementary file 5 — Supplementary Material 5. [file 41065_2025_607_MOESM5_ESM.pdf]
