# Supplementary material for: Uncovering new MicroRNAs linked to acute pancreatitis: zeroing in on the protective effect
Source: Hereditas. 2025 Dec 29;162:238. doi: 10.1186/s41065-025-00607-0 (PMC12751974; doi:10.1186/s41065-025-00607-0)
Supplement: Supplementary file 3 — Supplementary Material 3: Supplementary Table S2. Mendelian randomization analysis. [file 41065_2025_607_MOESM3_ESM.docx]

**Supplementary Table S2.** Mendelian randomization analysis.

| Exposure | Outcome | Method | n SNP | Beta | SE | P-value | OR | OR_lci95 | OR_uci95 |
| --- | --- | --- | --- | --- | --- | --- | --- | --- | --- |
| miR-4649-5p | AP | IVW | 8 | 0.136 | 0.065 | 0.036 | 1.146 | 1.009 | 1.301 |
| miR-493-5p |  |  | 8 | -0.129 | 0.066 | 0.0498 | 0.879 | 0.773 | 0.999 |
| miR-8061 |  |  | 11 | -0.086 | 0.042 | 0.039 | 0.918 | 0.845 | 0.996 |
| miR-6769a-5p |  |  | 13 | 0.139 | 0.067 | 0.039 | 1.149 | 1.007 | 1.310 |
| miR-573 |  |  | 7 | -0.095 | 0.047 | 0.045 | 0.909 | 0.829 | 0.998 |
| miR-147b |  |  | 10 | 0.147 | 0.055 | 0.007 | 1.159 | 1.040 | 1.291 |
| miR-219a-2-3p |  |  | 8 | 0.119 | 0.047 | 0.011 | 1.126 | 1.027 | 1.235 |
| miR-4455 |  |  | 11 | 0.091 | 0.046 | 0.046 | 1.096 | 1.001 | 1.198 |
| miR-6824-5p |  |  | 8 | 0.196 | 0.082 | 0.016 | 1.217 | 1.037 | 1.428 |
| miR-3662 |  |  | 8 | 0.154 | 0.074 | 0.038 | 1.166 | 1.008 | 1.349 |
| miR-5197-5p |  |  | 9 | 0.119 | 0.049 | 0.015 | 1.126 | 1.024 | 1.239 |
| miR-135a-5p |  |  | 4 | 0.151 | 0.073 | 0.037 | 1.164 | 1.009 | 1.342 |
| miR-6726-3p |  |  | 5 | -0.145 | 0.067 | 0.030 | 0.865 | 0.759 | 0.986 |
| miR-607 |  |  | 10 | 0.069 | 0.035 | 0.049 | 1.071 | 1.0002 | 1.147 |
| miR-600 |  |  | 17 | -0.087 | 0.030 | 0.004 | 0.917 | 0.865 | 0.972 |
| miR-1913 |  |  | 9 | 0.264 | 0.093 | 0.005 | 1.303 | 1.085 | 1.564 |
| miR-6750-3p |  |  | 7 | -0.148 | 0.065 | 0.022 | 0.862 | 0.759 | 0.979 |
| miR-6737-5p |  |  | 10 | -0.098 | 0.046 | 0.033 | 0.906 | 0.828 | 0.992 |
| miR-337-5p |  |  | 7 | -0.190 | 0.087 | 0.029 | 0.827 | 0.697 | 0.981 |
| miR-1277-5p |  |  | 5 | 0.121 | 0.052 | 0.021 | 1.129 | 1.019 | 1.251 |
| miR-205-5p |  |  | 40 | 0.044 | 0.014 | 0.002 | 1.045 | 1.016 | 1.074 |
| miR-4798-5p |  |  | 11 | 0.147 | 0.066 | 0.025 | 1.158 | 1.019 | 1.317 |
| miR-9-3p |  |  | 11 | 0.105 | 0.049 | 0.034 | 1.110 | 1.008 | 1.223 |
| miR-4291 |  |  | 7 | 0.374 | 0.161 | 0.020 | 1.454 | 1.061 | 1.992 |
| miR-30c-2-3p |  |  | 5 | 0.178 | 0.090 | 0.0496 | 1.194 | 1.0003 | 1.426 |
| miR-502-5p |  |  | 8 | -0.162 | 0.069 | 0.019 | 0.850 | 0.742 | 0.974 |
| miR-99a-5p |  |  | 5 | 0.296 | 0.146 | 0.042 | 1.344 | 1.010 | 1.789 |
| miR-106a-5p |  |  | 11 | 0.363 | 0.134 | 0.007 | 1.438 | 1.106 | 1.870 |
| miR-3936 |  |  | 9 | -0.079 | 0.037 | 0.033 | 0.924 | 0.860 | 0.994 |
| miR-6130 |  |  | 9 | -0.158 | 0.057 | 0.006 | 0.854 | 0.764 | 0.955 |
| miR-4436b-3p |  |  | 11 | -0.114 | 0.052 | 0.027 | 0.893 | 0.807 | 0.987 |
| miR-937-3p |  |  | 11 | -0.165 | 0.070 | 0.018 | 0.848 | 0.739 | 0.972 |
| miR-5190 |  |  | 8 | 0.110 | 0.056 | 0.0496 | 1.116 | 1.0002 | 1.245 |
| miR-4747-3p |  |  | 20 | -0.086 | 0.032 | 0.006 | 0.917 | 0.862 | 0.976 |
| miR-4668-5p |  |  | 6 | -0.204 | 0.103 | 0.047 | 0.815 | 0.666 | 0.998 |
| miR-4527 |  |  | 9 | 0.157 | 0.057 | 0.006 | 1.170 | 1.045 | 1.309 |
| miR-504-3p |  |  | 8 | 0.148 | 0.064 | 0.021 | 1.160 | 1.022 | 1.316 |
| miR-545-3p |  |  | 10 | 0.112 | 0.057 | 0.0499 | 1.119 | 1.0001 | 1.252 |
| miR-219b-3p |  |  | 8 | -0.109 | 0.039 | 0.005 | 0.897 | 0.831 | 0.967 |
| miR-551a |  |  | 8 | 0.152 | 0.065 | 0.018 | 1.165 | 1.026 | 1.322 |
| miR-4328 |  |  | 6 | -0.107 | 0.047 | 0.024 | 0.899 | 0.820 | 0.986 |
| miR-4483 |  |  | 10 | -0.146 | 0.047 | 0.002 | 0.864 | 0.788 | 0.948 |
| miR-450b-5p |  |  | 11 | -0.106 | 0.049 | 0.031 | 0.899 | 0.817 | 0.990 |
| miR-5002-5p |  |  | 9 | -0.117 | 0.059 | 0.047 | 0.890 | 0.793 | 0.998 |
| miR-4317 |  |  | 6 | 0.187 | 0.064 | 0.003 | 1.205 | 1.063 | 1.366 |
| miR-4675 |  |  | 11 | 0.089 | 0.038 | 0.021 | 1.093 | 1.013 | 1.178 |
| miR-5702 |  |  | 9 | 0.103 | 0.042 | 0.014 | 1.108 | 1.021 | 1.202 |
| miR-3692-3p |  |  | 7 | 0.180 | 0.058 | 0.002 | 1.197 | 1.069 | 1.341 |
| miR-3147 |  |  | 6 | 0.184 | 0.080 | 0.022 | 1.202 | 1.027 | 1.406 |
| miR-376c-3p |  |  | 8 | 0.218 | 0.111 | 0.049 | 1.243 | 1.001 | 1.545 |
| miR-3907 |  |  | 8 | 0.196 | 0.086 | 0.022 | 1.217 | 1.029 | 1.439 |
| miR-4761-5p |  |  | 15 | -0.078 | 0.040 | 0.049 | 0.925 | 0.855 | 0.999 |
| miR-183-3p |  |  | 9 | 0.122 | 0.046 | 0.008 | 1.130 | 1.032 | 1.237 |
| miR-27b-3p |  |  | 4 | -0.279 | 0.122 | 0.023 | 0.757 | 0.596 | 0.962 |
| miR-4322 |  |  | 5 | 0.171 | 0.081 | 0.033 | 1.187 | 1.014 | 1.390 |
| miR-193a-5p |  |  | 7 | -0.265 | 0.109 | 0.015 | 0.767 | 0.619 | 0.950 |
| miR-191-3p |  |  | 12 | 0.074 | 0.036 | 0.039 | 1.077 | 1.004 | 1.156 |
| miR-6787-5p |  |  | 14 | 0.137 | 0.050 | 0.006 | 1.147 | 1.041 | 1.264 |
| miR-523-3p |  |  | 12 | 0.062 | 0.032 | 0.048 | 1.064 | 1.001 | 1.132 |
| miR-193b-5p |  |  | 6 | -0.156 | 0.073 | 0.033 | 0.855 | 0.741 | 0.987 |
| miR-514b-5p |  |  | 13 | -0.169 | 0.049 | 0.001 | 0.845 | 0.767 | 0.930 |
| miR-1299 |  |  | 16 | 0.137 | 0.054 | 0.012 | 1.147 | 1.031 | 1.276 |
| miR-604 |  |  | 19 | 0.069 | 0.033 | 0.035 | 1.072 | 1.005 | 1.143 |
| miR-6728-3p |  |  | 9 | 0.125 | 0.057 | 0.030 | 1.133 | 1.012 | 1.267 |
| miR-106a-3p |  |  | 13 | 0.072 | 0.034 | 0.035 | 1.075 | 1.005 | 1.149 |
| miR-4273 |  |  | 8 | 0.088 | 0.044 | 0.046 | 1.092 | 1.002 | 1.191 |
| AP | miR-6769a-5p |  | 5 | -0.203 | 0.092 | 0.027 | 0.816 | 0.681 | 0.977 |
| AP | miR-106a-5p |  | 5 | -0.074 | 0.029 | 0.011 | 0.929 | 0.878 | 0.983 |
| AP | miR-193a-5p |  | 5 | 0.176 | 0.062 | 0.004 | 1.192 | 1.056 | 1.345 |
| AP | miR-4455 |  | 5 | -0.238 | 0.111 | 0.031 | 0.788 | 0.635 | 0.979 |

AP: acute pancreatitis; IVW: inverse variance weighted.
