# Supplementary material for: Uncovering new MicroRNAs linked to acute pancreatitis: zeroing in on the protective effect
Source: Hereditas. 2025 Dec 29;162:238. doi: 10.1186/s41065-025-00607-0 (PMC12751974; doi:10.1186/s41065-025-00607-0)
Supplement: Supplementary file 4 — Supplementary Material 4: Supplementary Table S3. Sensitivity analysis for the causal association between miRNAs and AP. [file 41065_2025_607_MOESM4_ESM.docx]

**Supplementary Table S3.** Sensitivity analysis for the causal association between miRNAs and AP.

| **MicroRNA** | **Heterogeneity** | | **Pleiotropy** | |
| --- | --- | --- | --- | --- |
|  | **Egger intercept** | **P-value** | **Q(I^2^)** | **P-value** |
| miR-4649-5p | 0.003 | 0.927 | 0 | 0.777 |
| miR-493-5p | 0.014 | 0.442 | 0 | 0.628 |
| miR-8061 | 0.023 | 0.349 | 0 | 0.499 |
| miR-6769a-5p | -0.022 | 0.328 | 26.424% | 0.177 |
| miR-573 | 0.014 | 0.554 | 0 | 0.924 |
| miR-147b | 0.027 | 0.180 | 0 | 0.758 |
| miR-219a-2-3p | 0.016 | 0.518 | 0 | 0.842 |
| miR-4455 | 0.004 | 0.826 | 32.187% | 0.142 |
| miR-6824-5p | 0.030 | 0.163 | 43.947% | 0.086 |
| miR-3662 | -0.055 | 0.164 | 41.099% | 0.104 |
| miR-5197-5p | 0.009 | 0.583 | 0 | 0.781 |
| miR-135a-5p | -0.026 | 0.579 | 0 | 0.425 |
| miR-6726-3p | 0.018 | 0.438 | 0 | 0.675 |
| miR-607 | 0.013 | 0.427 | 5.668% | 0.389 |
| miR-600 | -0.005 | 0.699 | 0 | 0.656 |
| miR-1913 | 0.005 | 0.822 | 0 | 0.491 |
| miR-6750-3p | 0.013 | 0.676 | 0 | 0.629 |
| miR-6737-5p | 0.017 | 0.351 | 0 | 0.650 |
| miR-337-5p | 0.000 | 0.984 | 0 | 0.510 |
| miR-1277-5p | 0.015 | 0.523 | 0 | 0.753 |
| miR-205-5p | -0.004 | 0.771 | 0 | 0.946 |
| miR-4798-5p | -0.040 | 0.074 | 15.767% | 0.294 |
| miR-9-3p | -0.020 | 0.340 | 0 | 0.741 |
| miR-4291 | 0.009 | 0.731 | 0 | 0.922 |
| miR-30c-2-3p | -0.020 | 0.675 | 33.419% | 0.199 |
| miR-502-5p | -0.002 | 0.957 | 1.908% | 0.415 |
| miR-99a-5p | -0.018 | 0.585 | 0 | 0.676 |
| miR-106a-5p | 0.012 | 0.471 | 0 | 0.898 |
| miR-3936 | 0.027 | 0.199 | 0 | 0.644 |
| miR-6130 | -0.017 | 0.702 | 0 | 0.539 |
| miR-4436b-3p | -0.010 | 0.531 | 7.720% | 0.370 |
| miR-937-3p | -0.006 | 0.743 | 0 | 0.604 |
| miR-5190 | -0.018 | 0.615 | 0 | 0.857 |
| miR-4747-3p | -0.010 | 0.500 | 0 | 0.774 |
| miR-4668-5p | 0.005 | 0.937 | 48.721% | 0.083 |
| miR-4527 | 0.020 | 0.471 | 33.998% | 0.146 |
| miR-504-3p | -0.018 | 0.566 | 2.237% | 0.412 |
| miR-545-3p | -0.007 | 0.778 | 0 | 0.778 |
| miR-219b-3p | -0.015 | 0.468 | 0 | 0.458 |
| miR-551a | 0.007 | 0.817 | 0 | 0.474 |
| miR-4328 | 0.007 | 0.733 | 0 | 0.718 |
| miR-4483 | -0.004 | 0.791 | 0 | 0.972 |
| miR-450b-5p | -0.007 | 0.830 | 10.623% | 0.343 |
| miR-5002-5p | -0.004 | 0.880 | 0 | 0.936 |
| miR-4317 | 0.007 | 0.849 | 0 | 0.422 |
| miR-4675 | -0.014 | 0.358 | 0 | 0.957 |
| miR-5702 | 0.013 | 0.478 | 0 | 0.446 |
| miR-3692-3p | 0.002 | 0.922 | 0 | 0.833 |
| miR-3147 | -0.025 | 0.467 | 0 | 0.641 |
| miR-376c-3p | 0.012 | 0.527 | 0 | 0.907 |
| miR-3907 | -0.005 | 0.823 | 0 | 0.460 |
| miR-4761-5p | 0.001 | 0.966 | 1.585% | 0.433 |
| miR-183-3p | 0.022 | 0.341 | 0 | 0.771 |
| miR-27b-3p | -0.015 | 0.579 | 0 | 0.523 |
| miR-4322 | -0.041 | 0.316 | 0 | 0.792 |
| miR-193a-5p | 0.039 | 0.402 | 0 | 0.942 |
| miR-191-3p | -0.009 | 0.552 | 0 | 0.601 |
| miR-6787-5p | -0.003 | 0.837 | 0 | 0.542 |
| miR-523-3p | -0.060 | 0.062 | 14.486% | 0.302 |
| miR-193b-5p | 0.030 | 0.321 | 0 | 0.664 |
| miR-514b-5p | 0.008 | 0.669 | 15.485% | 0.288 |
| miR-1299 | -0.002 | 0.900 | 0 | 0.555 |
| miR-604 | -0.009 | 0.657 | 23.370% | 0.172 |
| miR-6728-3p | 0.010 | 0.611 | 0 | 0.512 |
| miR-106a-3p | -0.005 | 0.794 | 0 | 0.743 |
| miR-4273 | -0.018 | 0.747 | 47.781% | 0.063 |
| miR-6769a-5p | 0.031 | 0.414 | 41.38% | 0.145 |
| miR-106a-5p | -0.007 | 0.562 | 16.10% | 0.312 |
| miR-193a-5p | 0.018 | 0.496 | 6.80% | 0.368 |
| miR-4455 | -0.012 | 0.784 | 0 | 0.994 |
